# Supplementary material for: Redesigning regulatory components of quorum-sensing system for diverse metabolic control
Source: Nat Commun. 2022 Apr 21;13:2182. doi: 10.1038/s41467-022-29933-x (PMC9023504; doi:10.1038/s41467-022-29933-x)
Supplement: Supplementary file 1 — Supplementary Information [file 41467_2022_29933_MOESM1_ESM.pdf]

Redesigning regulatory components of quorum-sensing system for diverse metabolic control

Ge *et. al*

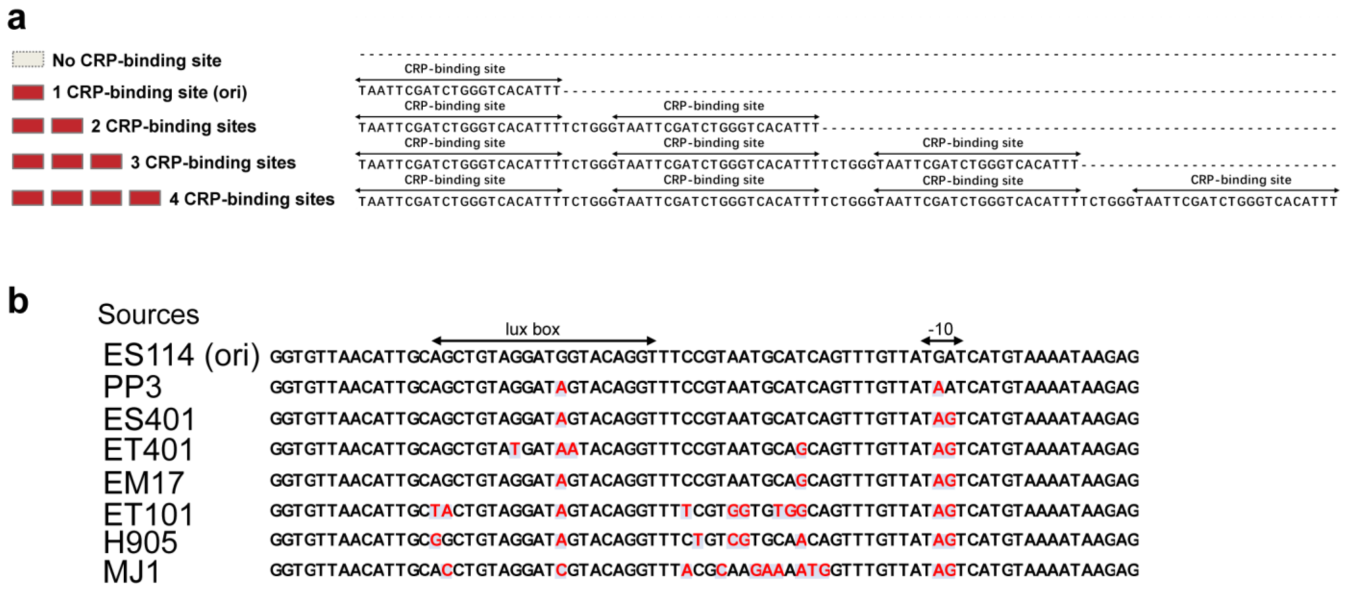

Supplementary Figure 1. The used CRP-binding sequence and lux box to -10 site sequences in this study. (a) The used CRP-binding site sequences in this study. (b) Source and sequence of the used “lux box” and “-10” site sequences. Red bases represent the mutated bases compared with the original sequence from ES114.

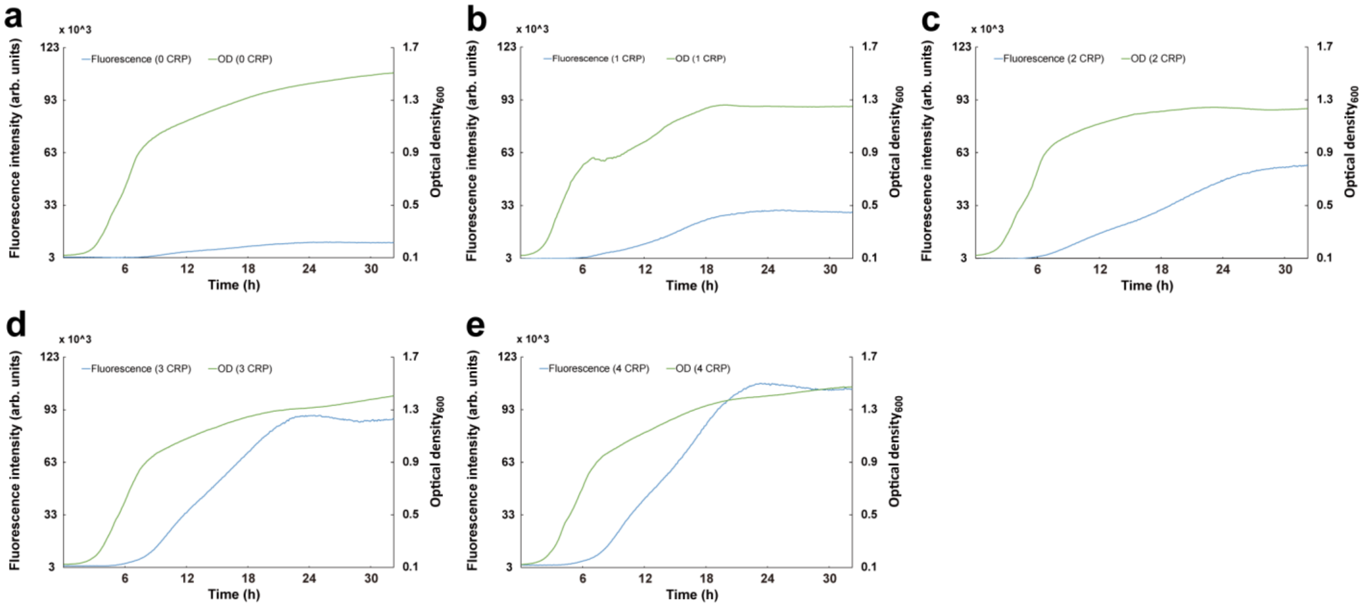

Supplementary Figure 2. The mCherry expression controlled by the P_{luxR} with different copies of CRP-binding site over a normal bacterial growth. (**a**) n=0; (**b**) n=1; (**c**) n=2; (**d**) n=3 (**e**) n=4. All data points are reported as mean from three independent experiments. Source data are provided as a Source Data file.

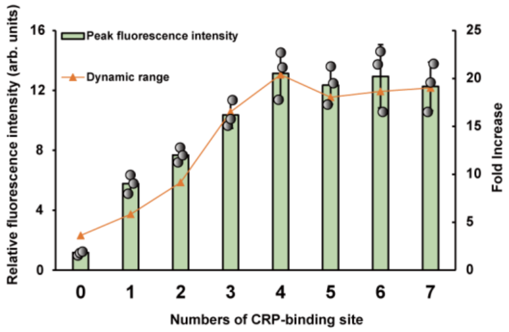

Supplementary Figure 3. The transcription levels the QS variant carrying different numbers of CRP-binding site (n=0,1,2,3,4,5,6,7). All data points are reported as mean ± s.d. from three independent experiments. Source data are provided as a Source Data file.

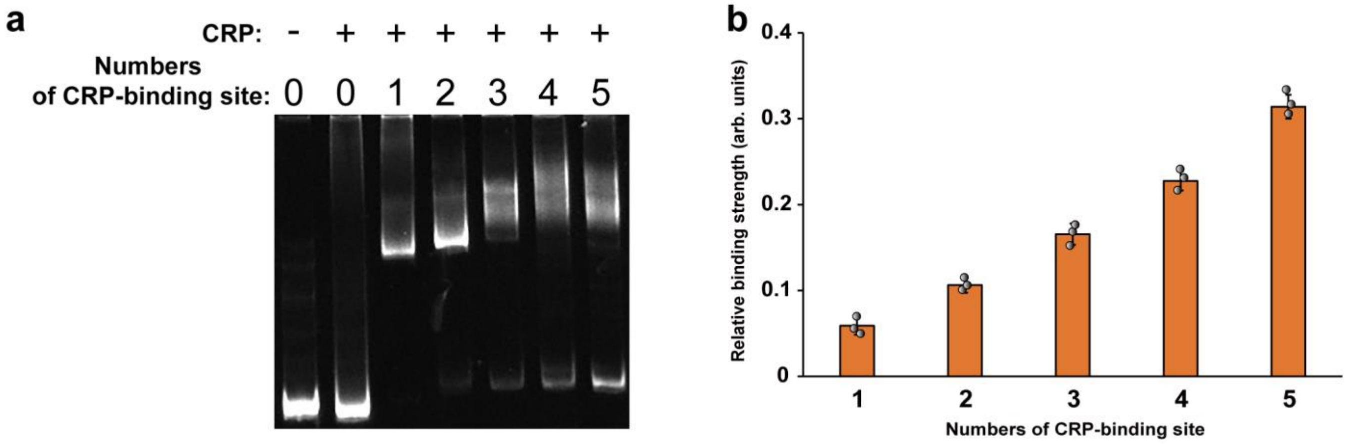

Supplementary Figure 4. The electrophoretic mobility shift assay for DNA probes with different numbers of CRP-binding site (n = 0, 1, 2, 3, 4, and 5) binding with CRP. (a) EMSA image. DNA probes at 50 nM; CRP at 250 nM. (b) Gray analysis based on the EMSA image. The relative binding strength was determined by the ratio of gray values of unbound DNA probe bands in the presence of CRP protein to the gray value of that in the absence of CRP protein. The gray analysis was performed by ImageJ bundled with 64-bit Java 1.8.0. All data points are reported as mean ± s.d. from three independent experiments. Source data are provided as a Source Data file.

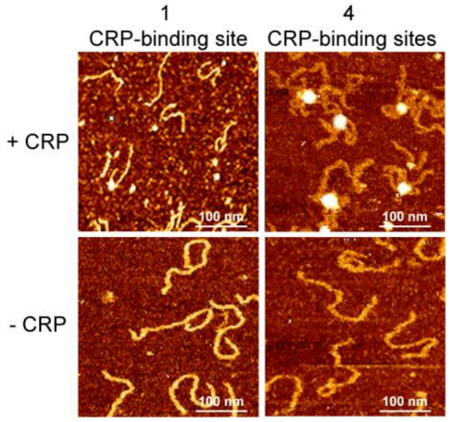

Supplementary Figure 5. Atomic force microscope observations of 1 or 4 CRP-binding sites DNA with or without CRP protein. The experiment was repeated independently for three times with similar results.

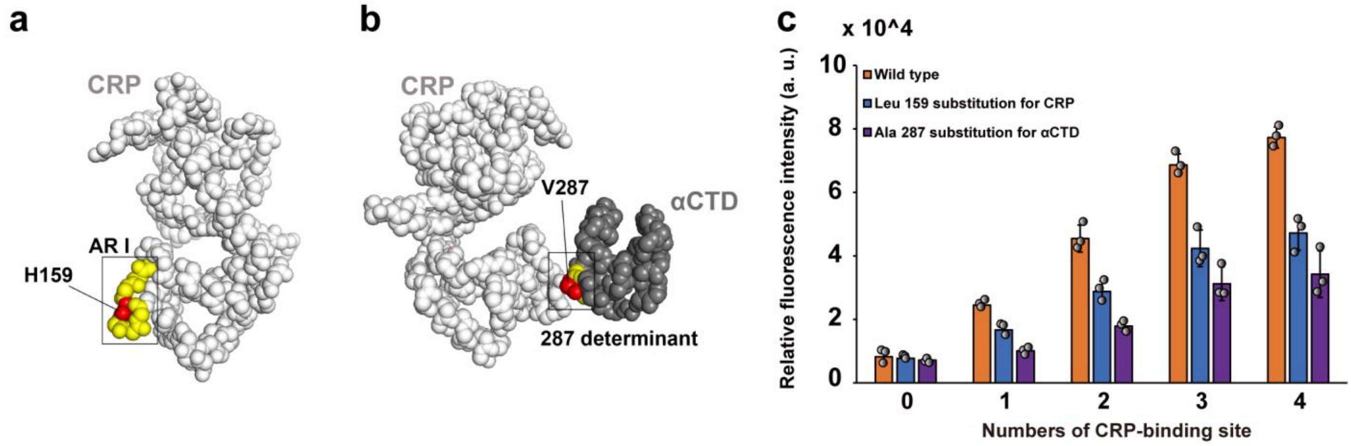

Supplementary Figure 6. The expression of P*luxR* after performing the Leu 159 substitution in CRP protein or the Ala 287 substitution in the αCTD of RNAP. These two substitutions are especially harmful to class I CRP-dependent activation, which were used to identify class I CRP-dependent activation. (**a**) Schematic diagram of the location of H159 in the CRP protein. AR 1, activating region 1. (**b**) Schematic diagram of the location of V278 in the αCTD of RNAP. (**c**) The transcription levels of P*luxR* after performing the Leu 159 substitution or the Ala 287 substitution. All data points are reported as mean ± s.d. from three independent experiments. Source data are provided as a Source Data file.

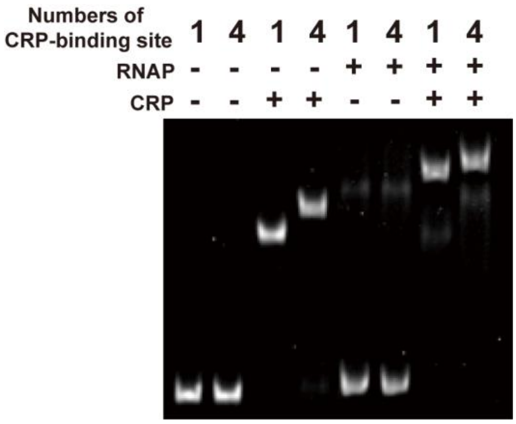

Supplementary Figure 7. The electrophoretic mobility shift assay for P_{luxR}-promoter probes with 1 or 4 CRP-binding sites. DNA probes at 50 nM; CRP at 250 nM; RNAP at 300 nM. The experiment was repeated independently for three times with similar results.

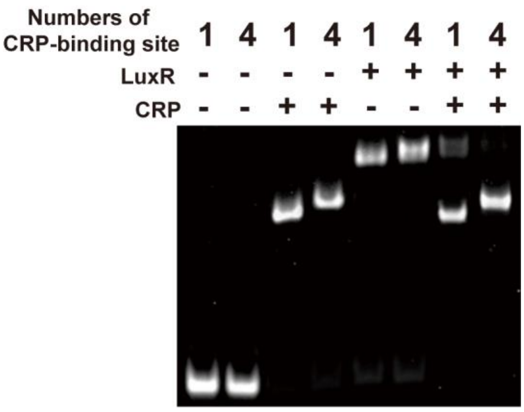

Supplementary Figure 8. The electrophoretic mobility shift assay for P*luxI*-promoter probes with 1 or 4 CRP-binding sites. DNA probes at 50 nM; CRP at 250 nM; LuxR at 300 nM. The experiment was repeated independently for three times with similar results.

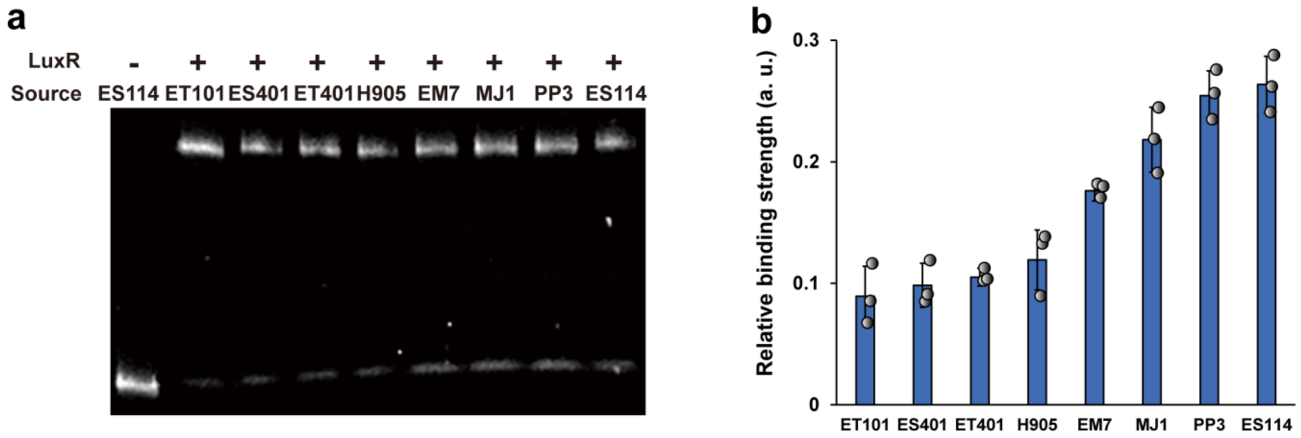

Supplementary Figure 9. The electrophoretic mobility shift assay for QS systems harboring different sources of lux box to -10 site sequence. (**a**) EMSA image. DNA probes at 20 nM; LuxR at 300 nM; RNAP at 300 nM. (**b**) Gray analysis based on the EMSA image. The relative binding strength was determined by the ratio of gray values of unbound DNA probe bands in the presence of LuxR protein to the gray value of that in the absence of LuxR protein. The gray analysis was performed by ImageJ bundled with 64-bit Java 1.8.0. All data points are reported as mean ± s.d. from three independent experiments. Source data are provided as a Source Data file.

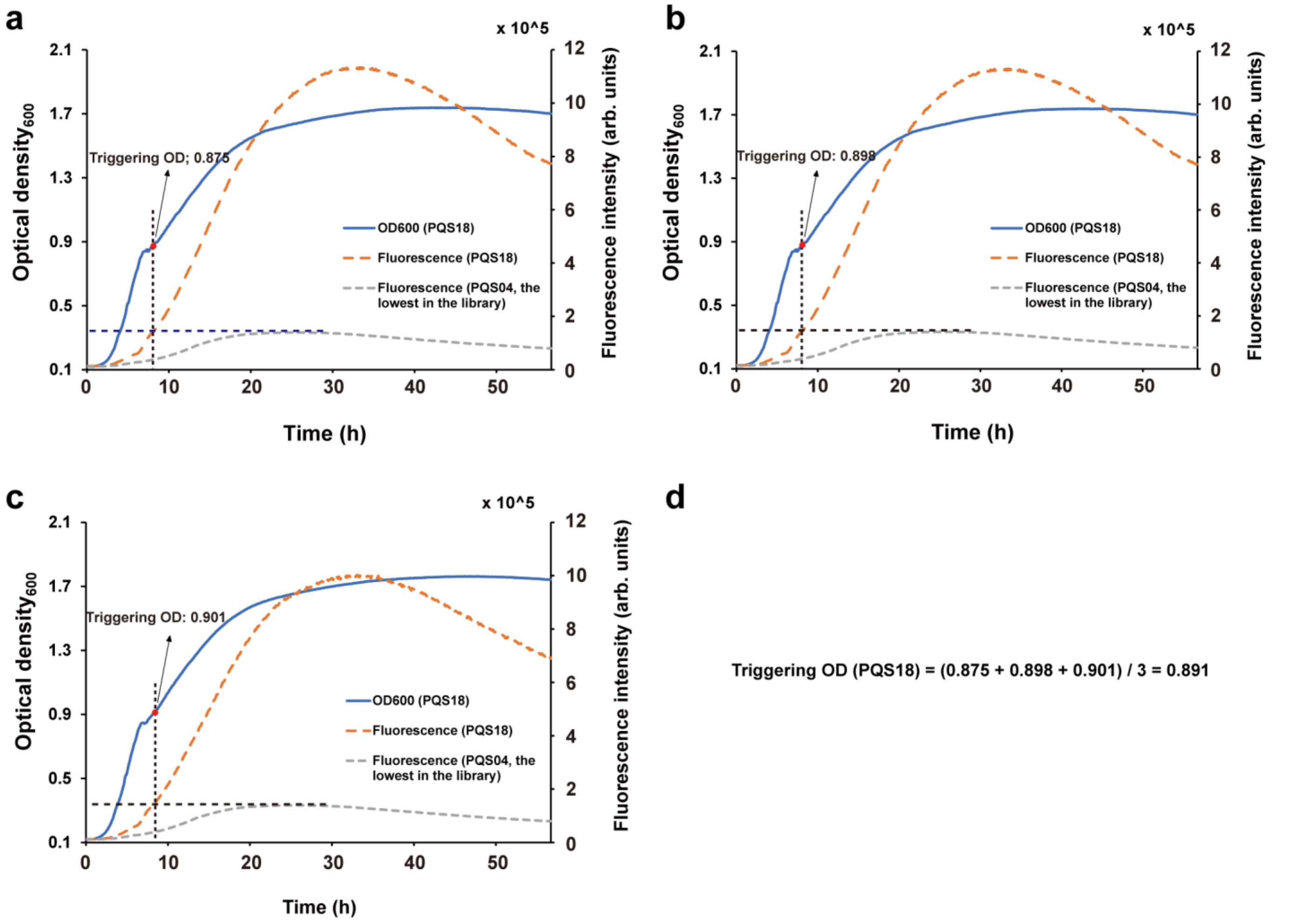

Supplementary Figure 10. Schematic diagram of determining the "triggering OD" using P_{QS18} as an example. (a)(b)(c) Three independent experiments for determining the "triggering OD" of P_{QS18}. (d) The calculation of "triggering OD" of P_{QS18}. Source data are provided as a Source Data file.

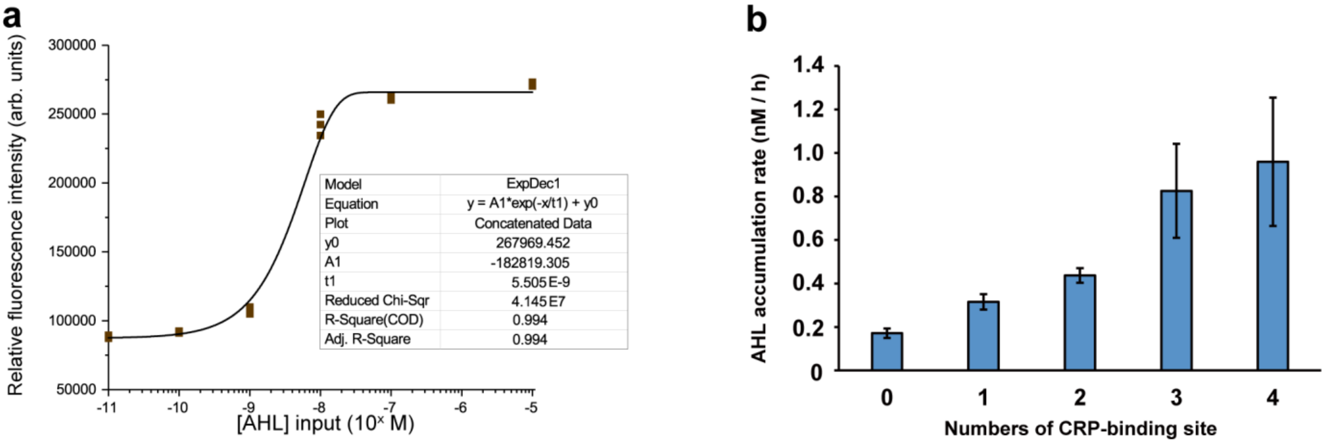

Supplementary Figure 11. AHL profiles for the QS systems harboring different numbers of CRP-binding site. (a) The corresponding relationship between AHL and relative fluorescence intensity. (b) The AHL accumulation rates of QS systems harboring different numbers of CRP-binding site (n=0,1,2,3,4). All data points are reported as mean ± s.d. from three independent experiments. Source data are provided as a Source Data file.

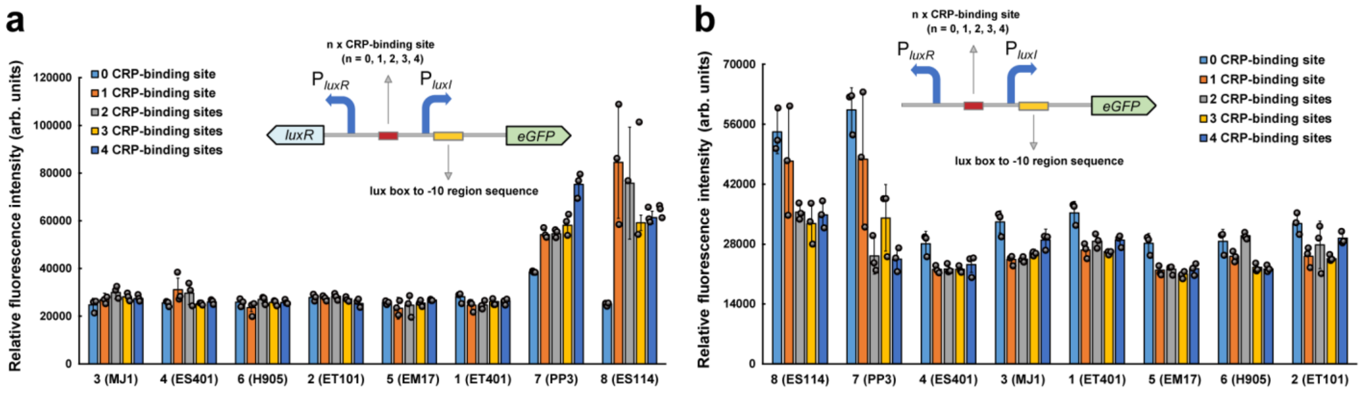

Supplementary Figure 12. Characterization of dynamic ranges and leaky expression of the QS variants in the library. (a) Fluorescence intensity of the QS variants in the absence of AHL. (b) Fluorescence intensity of the QS variants in the absence of AHL and LuxR. All data points are reported as mean ± s.d. from three independent experiments. Source data are provided as a Source Data file.

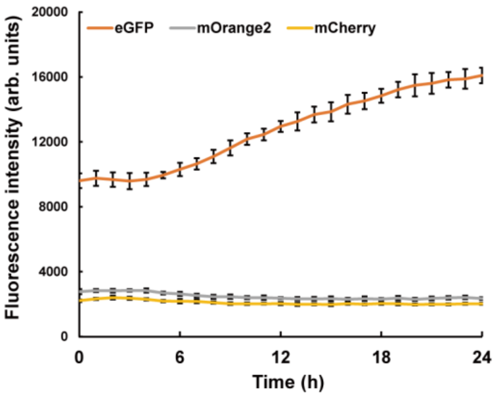

Supplementary Figure 13. The fluorescence value of the strains carrying no reporter proteins. All data points are reported as mean ± s.d. from three independent experiments. Source data are provided as a Source Data file.

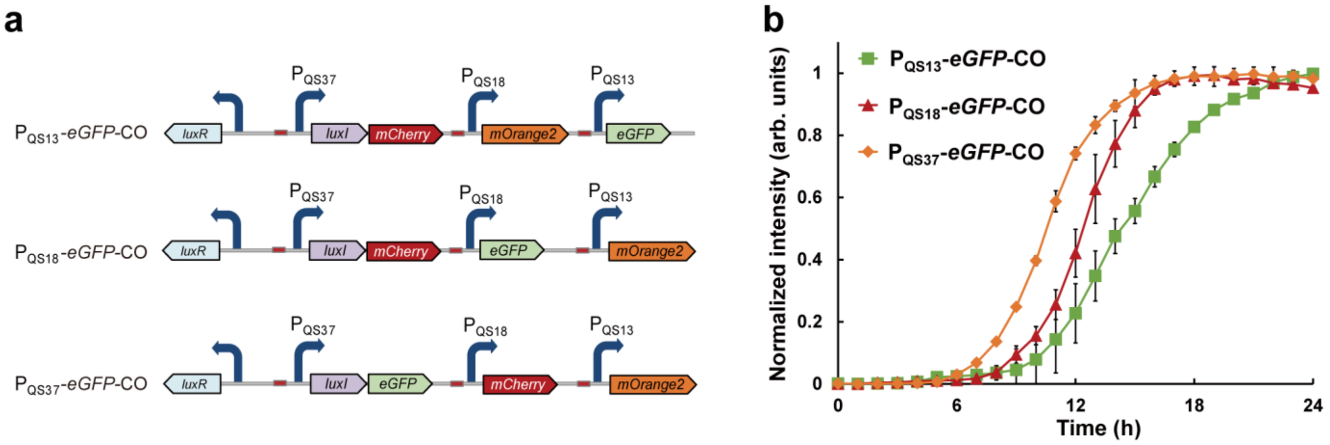

Supplementary Figure 14. Autonomous and simultaneous control of three different gene targets by QS variants. (a) Architecture of the QS circuits that simultaneously regulating the expression of eGFP, mCherry and mOrange2. Specifically, all promoters, PQS13, PQS18 and PQS37, expressed with eGFP, attempting to eliminating the potential impact of different protein maturation times. PQS13-eGFP-CO was used to present PQS13-controlled eGFP expression. PQS18-eGFP-CO was used to present PQS18-controlled eGFP expression. PQS37-eGFP-CO was used to present PQS37-controlled eGFP expression. (b) Fluorescence profiles for eGFP in the strains containing the designed QS circuit. For all strains, the fluorescent intensity of each time point was normalized by their corresponding peak fluorescence value. All data points are reported as mean ± s.d. from three independent experiments. Source data are provided as a Source Data file.

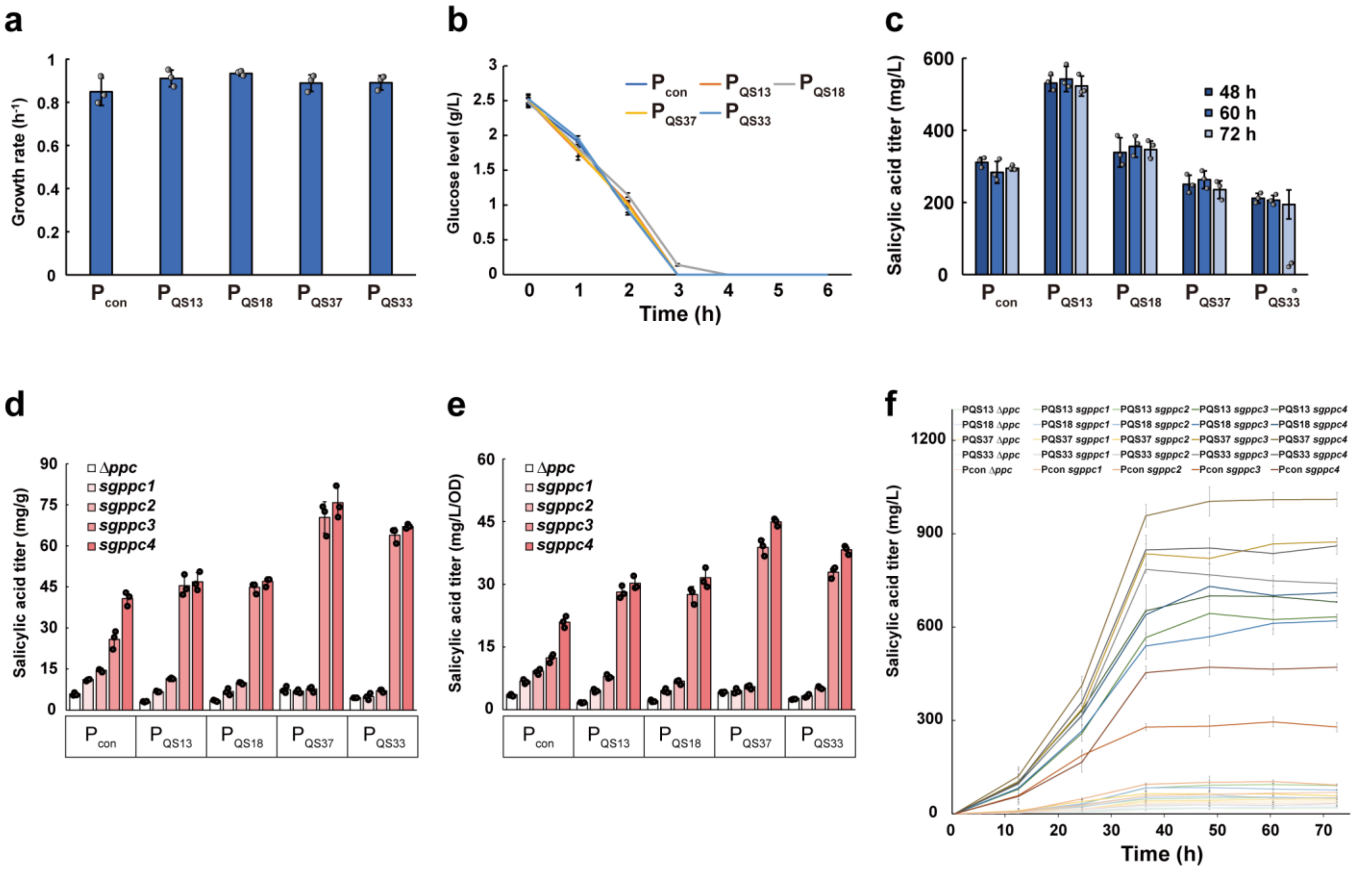

Supplementary Figure 15. Biosynthesis of SA using different QS circuits. (**a**) Growth rates of SA-producing strains. (**b**) Glucose consumption of SA-producing strains. (**c**) The SA titers of the SA-producing strains at different times. (**d**) The SA titers per consumed substrate. (**e**) The SA titers per OD_{600}. (**f**) The SA titers dynamics over cultivation time for all of SA-producing strains. All data points are reported as mean ± s.d. from three independent experiments. Source data are provided as a Source Data file.

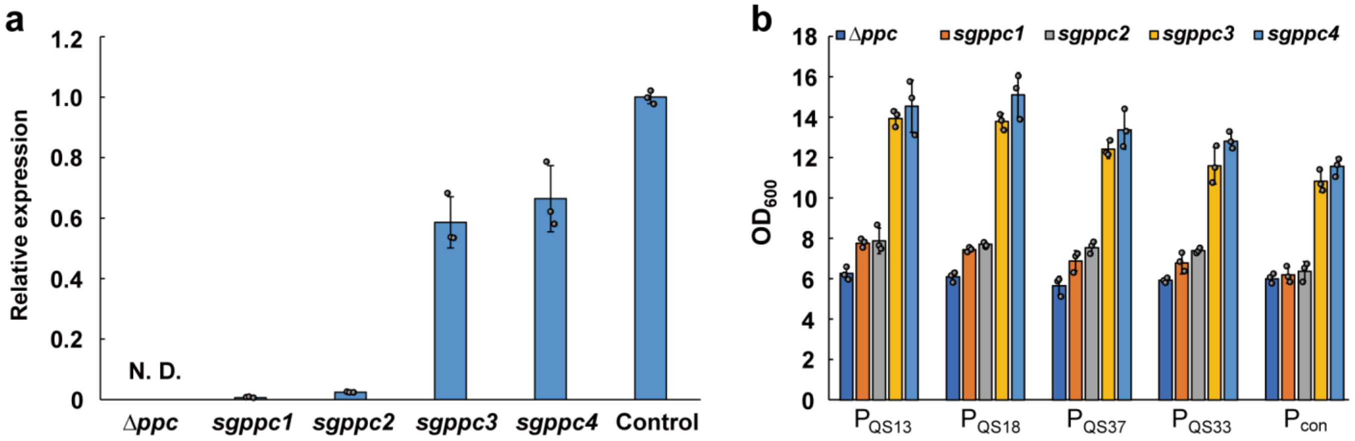

Supplementary Figure 16. The effect of simultaneous and dynamic up-regulation of *entC-pchB* and down-regulation of *ppc* on SA production. (a) The effect of different sgRNA targeting different locations of *ppc* operon on the transcriptional level of *ppc*. The expression level of *ppc* in all strains was normalized by that of the control strain. Control represents the transcriptional level of *ppc* in the strain without *ppc* down-regulation. N. D. = Not Detected. (b) Growth profiles for all SA-producing strains. All data points are reported as mean ± s.d. from three independent experiments. Source data are provided as a Source Data file.

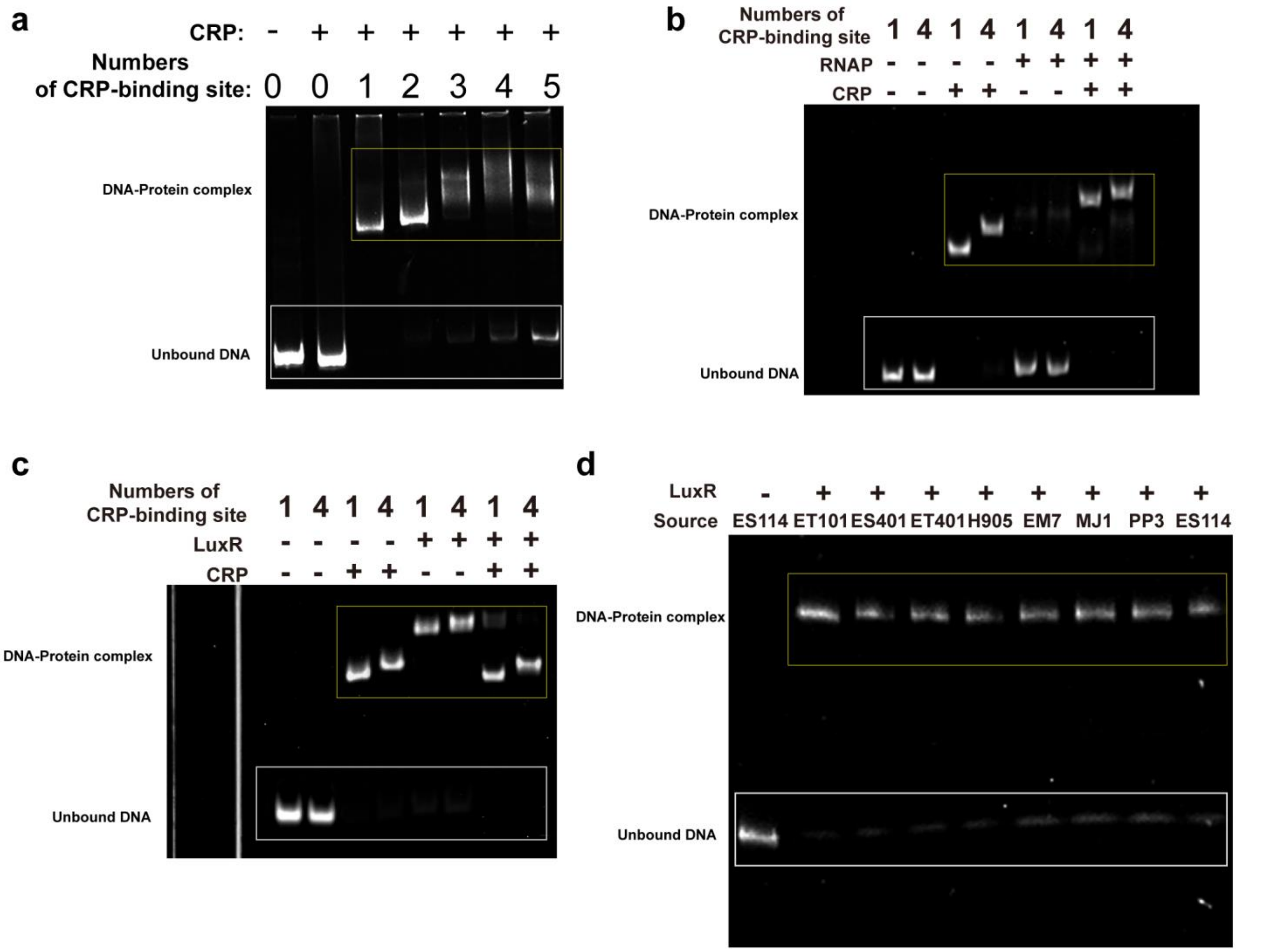

Supplementary Figure 17. Uncropped scans of gels included in figures. (a) EMSA gel of DNA probes with different numbers of CRP-binding site (n = 0, 1, 2, 3, 4, and 5) binding with CRP in Supplementary Figure 4. (b) EMSA gel of PluxR-promoter probes with 1 or 4 CRP-binding sites in Supplementary Figure 7. (c) EMSA gel of PluxI-promoter probes with 1 or 4 CRP-binding sites in Supplementary Figure 8. (d) EMSA gel of QS systems harboring different sources of lux box to -10 site sequence in Supplementary Figure 9. All data shown are representative of three independent experiments and at least three independent gels with similar results.
