# Supplementary material for: Redesigning regulatory components of quorum-sensing system for diverse metabolic control
Source: Nat Commun. 2022 Apr 21;13:2182. doi: 10.1038/s41467-022-29933-x (PMC9023504; doi:10.1038/s41467-022-29933-x)
Supplement: Supplementary file 3 — Description of Additional Supplementary Files [file 41467_2022_29933_MOESM3_ESM.pdf]

Description of Additional Supplementary Files

File Name: Supplementary Data 1
Description: Plasmids used in this study.

File Name: Supplementary Data 2
Description: Strains used in this study.

File Name: Supplementary Data 3
Description: Primers used in this study.
